# Outcomes of COVID-19 infection in patients with chronic lymphocytic leukemia: a systematic review and meta-analysis

**DOI:** 10.1590/1806-9282.20240322

**Published:** 2024-08-16

**Authors:** Mohammad Amin Akbarzadeh, Yosra Vaez-Gharamaleki, Farid Jahanshahlou, Alireza Ghaffari Bavil, Sina Hamzehzadeh, Sina Seifimansour, Alireza Rahimi-Mamaghani, Mohammad-Salar Hosseini

**Affiliations:** 1 Tabriz University of Medical Sciences, Research Center for Evidence-Based Medicine, Iranian EBM Center: A JBI Center of Excellence – Tabriz, Iran.; 2 Tabriz University of Medical Sciences, Hematology and Oncology Research Center – Tabriz, Iran.; 3 Tabriz University of Medical Sciences, Aging Research Institute, Research Center for Integrative Medicine in Aging – Tabriz, Iran.; 4 Tabriz University of Medical Sciences, School of Medicine, Department of Internal Medicine – Tabriz, Iran.; 5 Tabriz University of Medical Sciences, Medical Philosophy and History Research Center – Tabriz, Iran.; 6 Tabriz University of Medical Sciences, Liver and Gastrointestinal Diseases Research Center – Tabriz, Iran.; 7 Ardabil University of Medical Sciences, School of Medicine, Student Research Committee – Ardabil, Iran.; 8 Tabriz University of Medical Sciences, Clinical Research Development Unit of Tabriz Valiasr Hospital – Tabriz, Iran.

## INTRODUCTION

Since the beginning of the COVID-19 pandemic, complications of this disease among vulnerable populations have been a primary concern. Cancer patients are among the most vulnerable groups of patients owing to the compromised immune system and weakened overall health status^1^.

As a clonal malignancy of B lymphocytes, in which the abnormal monoclonal B lymphocytes are accumulated in the peripheral blood, bone marrow, and lymphoid tissues, chronic lymphocytic leukemia (CLL) results in compromised immune system by quantitative and qualitative deficiencies in lymphocyte populations vital for effective immune response and surveillance^2^. Additionally, cells exhibit aberrant expression of surface markers and altered cytokine signaling, resulting in impaired antigen presentation, defective cytotoxicity, and compromised humoral immunity^3^. Consequently, CLL patients show heightened susceptibility to bacterial, viral, and fungal infections, with increased morbidity and mortality attributable to infectious complications^4^.

Given the vulnerability of CLL patients and their susceptibility to COVID-19-related adverse events, this study aims to evaluate the impact of COVID-19 on the general conditions, prognosis, and clinical outcomes of patients with CLL.

## METHODS

This review was conducted in compliance with the recommendations of the Preferred Reporting Items for Systematic Reviews and Meta-Analysis (PRISMA), following an evidence-based research question, defined to address the impact of COVID-19 infection on clinical outcomes of CLL patients^5^. The study protocol is registered in the International Prospective Register of Systematic Reviews (PROSPERO, CRD42021244712).

### Search strategy

Medline (via PubMed), Scopus, Web of Science, and Cochrane Library were systematically searched using the free keywords and MeSH terms related to COVID-19, SARS-CoV-2, and Chronic Lymphocytic Leukemia, with no limitation in language and date of publication till the end of February 2024. To have a complete pool of related studies, the reference lists of studies were also assessed.

### Inclusion criteria and study selection

Two independent researchers screened the results by title, abstract, and full-text articles. All types of clinical studies were included if adult patients with CLL, small lymphocytic leukemia, or monoclonal B-cell lymphocytosis, and a confirmed diagnosis of COVID-19 were present. Studies with insufficient sample size, animal studies, reviews, consensus papers, and practical guidelines were excluded.

### Quality assessment and statistical analyses

Based on the type of the study, the remaining records were critically appraised by two independent researchers using the proper JBI Critical Appraisal Tools. CMA v2.0 was used to pool the data. A high degree of heterogeneity was defined as p value<0.05 or I^2^>50%, where a random-effect model would be used for pooling.

## RESULTS

**Figure 1:**
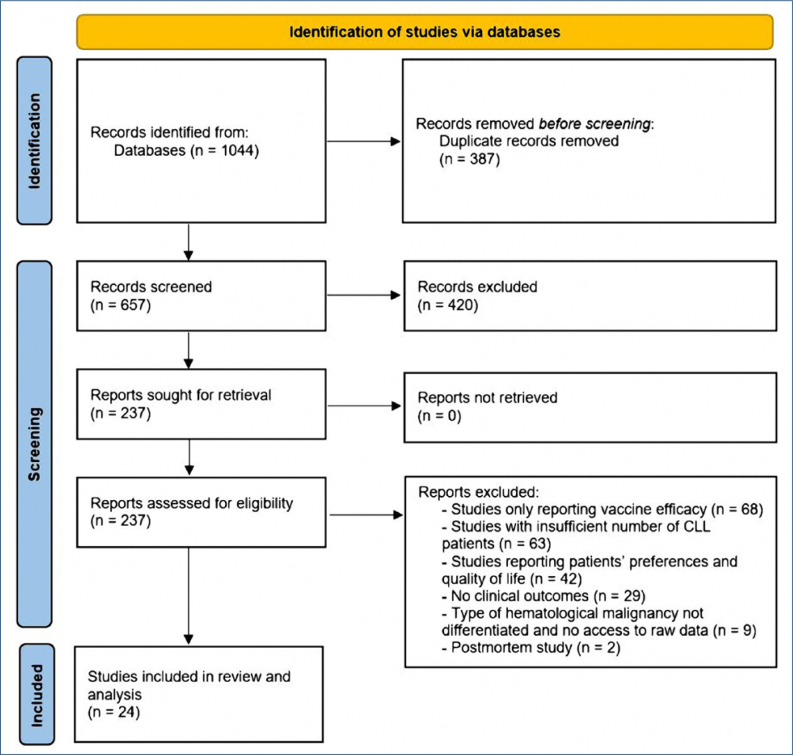
Preferred Reporting Items for Systematic Reviews and Meta-Analysis flow diagram of the study.

From the 1,044 search results, 387 duplicate records were removed. After screening the records, 24 were selected based on compatibility with the inclusion criteria (Figure 1). Of the 24 studies, 4 were flagged as high in risk of bias, but no study was removed from the pool.

### Patient characteristics

A total of 24 articles, reporting the results of 25 studies, with 7,091 patient records were included^6–29^. Most studies reported a median age of 68–72 years at the time of COVID-19 diagnosis (Table 1). Of the included patients, 64.6% were male, 39.82% were treatment-naïve, and 37.96% were under treatment at the time of COVID-19 diagnosis, most common with Bruton's tyrosine kinase inhibitor (59.34%).

**Table 1:** Characteristics of the included studies.

Authors	Year	Country	Number of CLL patients			Age (median)	Hospitalized	ICU admission	Deaths
			Total	Male	Female				
Aleshina et al.^6^	2023	Russia	45	N	N	N	45	18	14
Antic et al.^7^	2022	International	793	551	242	69 (IQR: 61–77)	593	162	N
Autore et al.^8^	2023	Italy	104	72	32	69 (IQR: 67–72)	N	N	10
Blixt et al.^9^	2022	Sweden	60	39	21	71 (range: 43–97)	46	11	14
Bronstein et al.^10^	2023	Israel	128	91	37	72 (IQR: 64–78)	34	N	6
Chatzikonstantinou et al.^11^	2021	International	941	628	313	69 (IQR: 61–77)	695	177	257
Cuneo et al.^12^	2021	Italy	494	N	N	324 patients >65 years	307	N	122
Glenthøj et al.^13^	2021	Denmark	31	19	12	(mean ± SD: 67.0 ± 16.7)	25	6	6
Kochneva et al.^14^	2022	Russia	136	84	52	66 (range: 42–90)	119	30	34
Mato et al.^15^	2020	International	198	125	73	70.5 (range: 38–98)	178	68	66
Mato et al.^16^	2023	USA	50	N	N	67 (range: 43–86)	28	N	14
Merli et al.^17^	2023	Italy	256	155	101	70 (IQR: 38–94)	176	50	77
Muntañola et al.^18^	2020	Spain	165	112	53	73 (range: 37–94)	152	47	45
Niemann et al.^19^	2022	Denmark	793	487	306	72 (IQR: 64–78)	105	13	37
Niemann et al.^20^	2022	International	67	N	N	69 (range: 43–90)	47	N	20
Puła et al.^21^	2022	Poland	188	119	69	68 (range: 37–87)	111	25	50
Roeker et al.^22^	2020	USA	30	22	8	65 (range: 41–82)	19	N	4
Roeker et al.^23^	2020	International	281	188	93	72 (range: 37–94)	281	56	85
Roeker et al.^23^	2020	International	130	83	47	68 (range: 41–98)	130	63	44
Scarfò et al.^24^	2020	International	190	126	64	72 (range: 48–94)	169	39	56
Šimkovič et al.^25^	2023	Czech Republic	341	237	104	69 (range: 39–92)	206	72	95
Stahl et al.^26^	2021	USA	25	N	N	N	N	N	2
Tekinalp et al.^27^	2022	Turkey	50	23	27	73 (range: 31–93)	50	36	13
Trajkova et al.^28^	2022	Republic of Macedonia	55	44	11	65	N	N	26
Visentin et al.^29^	2023	International	1,540	998	542	69 (IQR: 62–77)	1,007	240	368

CLL: chronic lymphocytic leukemia; ICU: intensive care unit; IQR: interquartile range; N: not reported.

### Hospitalization and mortality

**Figure 2:**
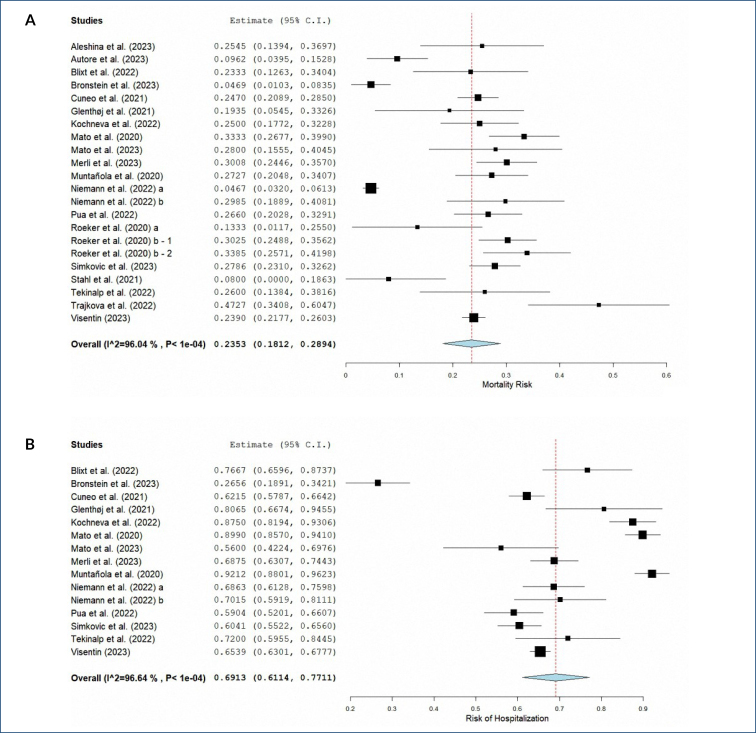
Pooled analysis for risk of mortality (a) and hospitalization (b) in chronic lymphocytic leukemia patients with COVID-19 infection.

Data regarding COVID-19-related mortality with a follow-up time of at least 1 month were pooled. Twenty-two independent studies with 5,167 patients had available data for COVID-19-related mortality in CLL patients. The pooled mortality risk was estimated as 23.53% (95%CI: 18.12–28.94%), with a considerable heterogeneity (I^2^=96.04%, p value<0.01). The pooled risk of hospitalization was estimated as 69.13% (95%C I: 61.14–77.11%), also with a high heterogeneity (I^2^=96.64%, p value<0.01) (Figure 2). A 28.27% (95%CI: 23.55–33.52%) risk of admission to intensive care unit was estimated among hospitalized patients. The included studies reported decreased hospitalization rates through the pandemic; however, sufficient data were unavailable to perform a pooled analysis. An overall mortality of 33.3% (95%CI: 29.72–37.16%) was estimated among the hospitalized patients. No statistically significant difference was detected in the pooled risk of hospitalization between male and female patients within the available data (OR=1.19, 95%CI: 0.50–2.84, p value: 0.68).

### Underlying conditions and medical history

The pooled risks of hospitalization and death in patients with a history of hypertension were 90.09% (95%CI: 82.56–94.58%) and 28.18% (95%CI: 14.09–48.43%), and in patients with a history of diabetes mellitus were 81.17% (95%CI: 71.69–88%) and 30.4% (95%CI: 20.45–42.73%), respectively. The pooled risks of hospitalization and death in patients with a history of coronary artery disease and/or cardiac failure were 78.43% (95%CI: 54.3–91.75%) and 32.03% (95%CI: 19.93–47.13%), respectively. The risks of hospitalization and mortality were not reported in a sufficient number of studies to report a pooled ratio for patients with a history of arrhythmia, asthma, chronic obstructive pulmonary disease, and chronic kidney disease. CLL patients with active smoking were at an 81.18% (95%CI: 66.64–90.31%) risk of hospitalization and 38.58% (95%CI: 13.38–71.86%) mortality risk. Overall, 46.1% of the patients had a Cumulative Illness Rating Scale (CIRS) score over 6. Patients with CIRS>6 had an 87.73% (95%CI: 76.23–94.1%) risk of hospitalization and 34.75% (95%CI: 22.3–49.7%) risk of mortality.

A poor Eastern Cooperative Oncology Group (ECOG) performance status scale was significantly reported with an increased risk of COVID-19-related mortality. Also, age over 65 years at the time of COVID-19 diagnosis was significantly associated with higher hospitalization and mortality rates; however, these variables had insufficient data for pooling.

### Clinical presentation, symptoms, and complications

Fever, cough, dyspnea, and fatigue were the most common presentations in CLL patients with COVID-19 infection, with a prevalence of 74.30%, 60.14%, 48.05%, and 37.97%, respectively. Myalgia, diarrhea, and nausea/vomiting were the less common symptoms, with a prevalence of 15.52%, 13.46%, and 8.01%, respectively. From 1,142 available patient records, a pooled risk of disseminated intravascular coagulation /thrombosis of 11% (95%CI: 7.13–16.59%) was estimated for CLL patients as the main complication of COVID-19 infection.

Unlike other symptoms, fever at the time of COVID-19 diagnosis is suggested to be associated with hospitalization in the included studies (reported HR=2.07, 95%CI: 1.03–4.19, p value<0.05); however, available data were not sufficient for a pooled analysis. Anemia, thrombocytopenia, and elevated lactate dehydrogenase level at the initial assessment were statistically associated with adverse clinical outcomes and COVID-19-related mortality. One study (Visentin et al.) reported the WHO-defined post-COVID condition in CLL COVID-19 survivors, with a prevalence of 15.8%.

## DISCUSSION

Due to the global impact of the COVID-19 pandemic, cancer patients who contact the virus have experienced adverse outcomes. Recent data indicate a general susceptibility of individuals with hematological malignancies to SARS-CoV-2 infection, and, at the top of all, CLL, making them more prone to severe forms of COVID-19^30^.

The mortality rate in CLL patients varies based on different factors, including age, stage of the disease, overall health, the presence of underlying comorbidities, and the effectiveness of supportive treatments. In the context of COVID-19, CLL patients may face an increased risk of mortality due to the immunocompromised state associated with the disease and the systemic treatment. Previous studies have reported a case fatality rate of 32–47%^31,32^. CLL patients with symptomatic COVID-19 and requiring inpatient admission showed high mortality rates.

Advanced age is consistently associated with increased mortality in COVID-19 patients, and a similar trend has been observed among CLL patients as well. Aging is associated with a weakened immune system, limited physiological reserve, and organ failures. Underlying health conditions or comorbidities, such as cardiovascular disease, diabetes, or respiratory conditions, may lead to a higher rate of mortality in CLL patients with COVID-19. The severity of both CLL and COVID-19 plays a crucial role in this matter. Patients with advanced CLL may have compromised immune systems, making them more vulnerable to severe manifestations of COVID-19^33^.

Humoral and cellular immunity dysfunctions contribute to the heightened susceptibility. Antineoplastic CLL therapies, such as specific immunosuppressive or chemotherapeutic agents, may affect the patient's ability to mount an effective immune response against infections, possibly due to different impacts on immune system components. For instance, some studies suggest that CLL patients under ibrutinib are less likely to require hospitalization, suggesting diverse effects of anti-leukemic treatments^34^.

The COVID-19 pandemic has significantly affected the means, standards, and quality of care as the medical care has undergone major changes in adaptations during the pandemic in both oncology and nononcology care settings^35,36^. Further general and cancer-oriented studies are required to clarify these transforms and draw updated clinical conclusions for future practice^37^. Current review suggests that CLL patients, specifically symptomatic patients, suffer from higher mortality and morbidity due to COVID-19 regardless of their disease phase or treatment status. These results highlight the importance of understanding and addressing the specific challenges that CLL patients may encounter in the context of COVID-19 and future pandemics.

## CONCLUSION

CLL patients are at higher risk of COVID-19-related adverse outcomes, including hospitalization, need for intensive care, and mortality. Further population-based studies should be conducted to determine the role of underlying conditions, the status of antineoplastic treatment, and potential antiviral therapies on the clinical course of COVID-19 and post-COVID condition.
